# Does heterogeneity matter in the estimation of tumour budding and tumour stroma ratio in colon cancer?

**DOI:** 10.1186/s13000-018-0697-9

**Published:** 2018-03-20

**Authors:** Ann C. Eriksen, Johnnie B. Andersen, Jan Lindebjerg, René dePont Christensen, Torben F. Hansen, Sanne Kjær-Frifeldt, Flemming B. Sørensen

**Affiliations:** 1 Institute of Regional Health Research, University of Southern Denmark, Odense, Denmark; 2 Department of Pathology, Danish Colorectal Cancer Center South, Vejle Hospital, Beriderbakken 4, DK-7100 Vejle, Denmark; 3 Department of Clinical Medicine, Stereological Research Laboratory and University Institute of Pathology, Aarhus University, Nørrebrogade 44, 10G, DK-8000 Aarhus C, Denmark; 4 Visiopharm A/S, Hoersholm, Denmark; 5 Research Unit of General Practice, University of Southern Denmark, J.B. Winsløws Vej 9 A, 1st, DK-5000 Odense C, Denmark; 6 University Institute of Pathology, Aarhus University Hospital, PalleJuul-Jensen Boulevard 99, Entrance F, Plan 1, C 1.112, DK-8200 Aarhus N, Denmark

**Keywords:** Colon cancer, Heterogeneity, Tumour budding, Tumour stroma ratio

## Abstract

**Background:**

Tumour budding (TB) and Tumour Stroma Ratio (TSR) may be rewarding in the treatment stratification of patients with stage II colon cancer. However, lack of standardization may exclude these parameters from being used in a clinical setting. The purpose of this methodologic study was to compare stereology with semi-quantitative estimations of TSR, to investigate the intra-tumoural heterogeneity of TB and TSR, and to assess the intra- and inter-observer agreement.

**Methods:**

Three paraffin embedded tumour blocks, one of them representing the deepest invasive front, were selected from each of 43 patients treated for stage II colon cancer. TSR was estimated in H&E sections semi-quantitatively using conventional microscopy, and stereologically on scanned slides, using the newCAST stereology platform. TB was scored across 10 high power fields at the invasive front in cytokeratin AE1/AE3 stained sections.

**Results:**

Subjective, semi-quantitative estimates of TSR significantly correlated to the stereological estimates, with the best correlation found for sections with the deepest invasive tumour penetration (σ = 0.621, *p* < 0.001). Inter-observer agreement was moderate to substantial for both TB (Κappa = 0.46–0.73) and TSR (Κappa = 0.70–0.75).

The Intraclass correlation coefficient (ICC) for TSR varied from 0.322 based on stereological hotspot estimation to 0.648 for the semi-quantitative evaluation. For TB, ICC varied from 0.646 based on continuous data to 0.698 based on categorical data (cut-off: 10 buds). Thus, the intra-tumoural heterogeneity for both TB and the semi-quantitative estimation of TSR was low.

**Conclusion:**

We recommend using only one tissue section representing the deepest invasive tumour area for estimation of TSR. For TB we recommend using one tissue section; however due to low representation of high-budding tumours, results must be considered with caution.

**Electronic supplementary material:**

The online version of this article (10.1186/s13000-018-0697-9) contains supplementary material, which is available to authorized users.

## Background

Colon cancer (CC) is among the most frequent cancers in the Western World [1]. Survival is primarily correlated to the extension of the disease at the time of diagnosis. However, patients diagnosed with the same stage of disease often have markedly different outcomes [2]. This is a clinical challenge, especially in stage II disease, and new biomarkers are requested to select high-risk patients for adjuvant chemotherapy after intended curative surgery. In this context, the tumour microenvironment has been investigated in various settings and several studies have found *Tumour Stroma Ratio* (TSR) and *Tumour Budding* (TB) to provide prognostic information.

TSR is an estimate of the proportion of malignant epithelial cells and stroma, evaluated in a hematoxylin and eosin (H&E) stained tissue section, representing the deepest invasive area of the primary tumour [3]. High TSR (= stroma-low) is associated with significantly better overall survival (OS) and recurrence-free survival (RFS) compared to low TSR (= stroma-high). Several investigations have reported intra-tumoural stroma-epithelium ratio, or tumour stromal percentage, to be an independent prognostic marker of clinical relevance in CC [3–8]. These studies use different methodologies varying from simple visual, semi-quantitative estimation based on either conventional microscopy [3, 4] or digital pathology [5] to more objective morphometric methods [6, 8]. These various techniques have, to our knowledge not yet been compared.

Heterogeneity of TSR has been described in individual tumours [3, 6]. To compensate for this, Mesker et al. [3] recommended using the histologic section from the primary tumour with the highest T stage, as they documented the tumour slide with the deepest infiltration in the bowel wall to have the lowest fraction of adenocarcinoma cells (i.e.*,* highest stroma fraction). Heterogeneity of TSR in CC needs to be further investigated from the methodological point of view for future clinical, diagnostic implementation.

TB may reflect the *epithelial mesenchymal transition* (EMT) at the invasive tumour front and thus represent the cell-biological correlate of the tumour-stroma-interphase. Tumour buds are defined as single tumour cells, or clusters of up to 4 tumour cells, in the stroma at the invasive tumour margin [9]. Several studies have found TB to be a prognostic marker for both stage II colorectal cancer (CRC) [10–12] and for CC exclusively [13–15], and it has recently been incorporated into guidelines such as The College of American Pathologists Protocol (www.cap.org/cancerprotocols). Also, the International Tumor Budding Consensus Conference Group [16] strongly recommends TB to be included as a high-risk factor for stage II CRC, and thus clearly recommends evaluating TB; however, using H&E or immunohistochemical stained sections was an issue for disagreement in the group. Most studies use H&E stained sections [10, 11, 13–15], although cytokeratin-stained tissue sections may detect three to four times more tumour buds and has a higher reproducibility [17]. Recent studies have recommended this immunohistochemical approach for counting TB [12, 18]. Intra-tumoural heterogeneity of TB has only been sparsely touched in the scientific literature. To compensate for tumour heterogeneity, a 10-high-power fields (HPFs) scoring technique has been proposed [12, 18], but the method only accounts for heterogeneity within the selected section. Also, it is well known that CC is architecturally, molecularly, and biologically heterogeneous [19], and intra-tumoural heterogeneity may have significant impact on the interpretation of biomarkers [20]. This challenge has to our knowledge not been investigated for TB in CC.

The aim of this methodological study was to investigate the heterogeneity of TSR and TB in stage II CC, and to address the issue of whether the histologic section representing the deepest invasive tumour margin is representative of the whole tumour regarding TSR and TB. We compare semi-quantitative estimation of TSR by conventional microscopy with the quantitative gold standard represented by stereology. Finally, we provide data on intra- and inter-observer reproducibility.

## Methods

### Patients and tissue

Archival, formalin fixed, paraffin embedded tumour tissue samples from 43 consecutive patients, operated for stage II CC at the Department of Surgery, Vejle Hospital, Denmark in 2002, were retrieved. None of the patients had received preoperative chemo- or radiotherapy. The study population consisted of six mucinous adenocarcinomas and 37 adenocarcinomas (NOS). Mean age was 72.7 years (range 48–70). According to the 7th edition of American Joint Committee (AJCC) TNM classification, 34 of the tumours were classified as T3 and nine as T4. The total number of tumour-containing tissue blocks *per* patient varied from three to 24 (mean = 5.3). Histologic sections of 4 μm thickness were cut from all blocks (*N* = 229) and stained with H&E. Each tumour was represented by three sections.

Using a 2.5× or 5× objective the section representing the deepest invasive front of the tumour was selected. Two additional sections were selected using a random number table.

### Tumour stroma ratio

We estimated TSR in H&E stained sections by two different approaches: a) subjective, manual (semi-quantitative) by conventional microscopy, and b) stereology (quantitative), using computer assisted software.

***a)*** Using a 2,5× or 5× objective with field size area 78.5mm^2^ and 19.6 mm^2^, respectively, the invasive area with the highest representation of tumour stroma was selected. Subsequently, using a 10× objective with field size area 4.9 mm^2^ the part of the sample with the highest fraction of stroma (hot-spot sampling) was subjectively selected. Tumour cells were present at all borders of the image field (north-east-south-west) as described by Huijbers et al. [4]. The stroma percentage was estimated *per* microscopic field and scored into four groups (1: TSR > 75%, 2: 50% < TSR ≤ 75%, 3: 25% < TSR ≤ 50%, and 4: TSR ≤ 25%). Whenever a score was difficult to settle in the selected area, the decision was guided by the overall impression of the stromal fraction in the tumour. Areas with necrosis were avoided. In mucinous tumours, the area with mucin was visually excluded for the scoring. Major vascular structures and smooth muscle tissue were also visually excluded, whereas nerves, smaller vascular structures and lymphocytic infiltration were not excluded from the stromal compartment.

**Fig. 1:**
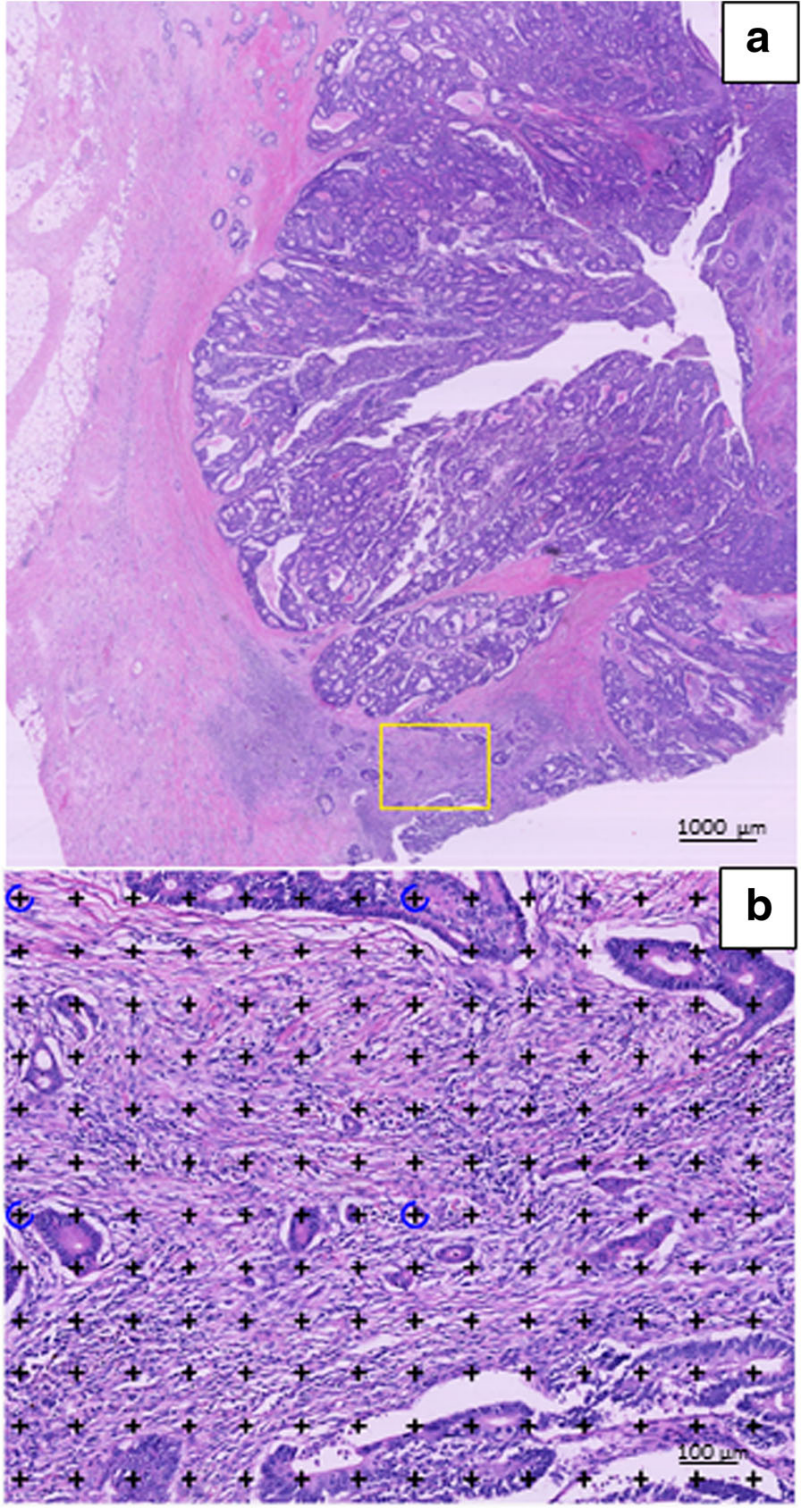
Stereological estimation of tumour stroma ratio. **a** The area with the highest stroma density (yellow frame) is subjectively selected at low magnification (2×). **b** At higher magnification (10×) a grid of 4 x (6 × 7) points is superimposed on the selected area. Points hitting either tumour cells or stroma are counted separately. Tumour cells must to be present at all borders of the visual field (north-east-south-west)

***b)*** The stereological analysis was performed to obtain unbiased, absolute estimates of the TSR, using the computer assisted stereology system newCAST (Visiopharm, Hoersholm, Denmark). All H&E stained sections were scanned at 40× magnification by a NanoZoomer XR scanner (Hamamatsu, Japan). The image format was NanoZoomer Digital Pathology Image (*.ndpi) with a resolution of 226 nm/pixel (112,389 dots *per* inch (DPI), i.e. 4.4 × 4.4 pixels/μm, corresponding to a final magnification of × 1.558). First, at low magnification (2×) we estimated TSR in the whole tumour area, which was manually outlined as the region of interest (ROI), and superimposed a grid with 5 × 5 points (25 points). We used systematic random, uniform sampling and a sample fraction of 100%. A point (i.e. a cross) was counted as tumour, whenever the upper right corner of a cross hit a viable tumour cell, and as stroma, whenever the upper right corner of a cross hit a stromal area. Points hitting areas of smooth muscle, adenoma, tumour lumen, necrosis, mucin, and large vessels were not counted. Subsequently, the area representing the highest density of stroma (the hotspot sampling) was investigated at × 10 magnification, using a grid with 4 groups and 6 × 7 points (42 points per group) superimposed on the selected area (Fig. 1), and points hitting tumour cells or stroma were counted as mentioned above. Tumour cells were required to be present at all borders of the image field (north-east-south-west) similar to the semi-quantitative method. The TSR was afterwards calculated as

$TSR=\frac{\sum {P}_{tumour}}{\sum {P}_{tumour+ stroma}}$*,* where P denotes points counted.

### Immunohistochemistry

Serial sections were cut from the selected tumour blocks (*N* = 129) and mounted on FLEX IHC Microscope Slides (K8020, Agilent DAKO products, Glostrup, Denmark). The pretreatment processes were performed using PT Link (DAKO). Heat-induced epitope retrieval was achieved with Envision Target Retrieval Solution (DAKO) at pH 9 and 97 °C for 20 min. Staining was performed using a DAKO Autostainer Link 48 (DAKO). Endogenous peroxidase activity was blocked by Envision FLEX Peroxidase-Blocking Reagent (DAKO). The primary antibody was mouse monoclonal cytokeratin AE1/AE3 (code M3515, DAKO) diluted 1:250 with Envision Flex antibody diluent (code S2022 DAKO). Primary antibody was incubated for 30 min. at room temperature, and for amplification Envision Flex+ Mouse (Linker) (DAKO) was used for 20 min. Bound antibodies were detected by Envision FLEX/HRP (DAKO) and visualised by Envision FLEX DAB (DAKO) with chromogen diluted in Envision Flex Substrate Buffer (DAKO). Meyer’s hematoxylin (Merck, Darmstadt, Germany) was used as counterstain and finally, the histological slides were cover slipped with Tissue-Tek PERTEX (Histolab Products AB, Göteborg, Sweden).

### Tumour budding

**Fig. 2:**
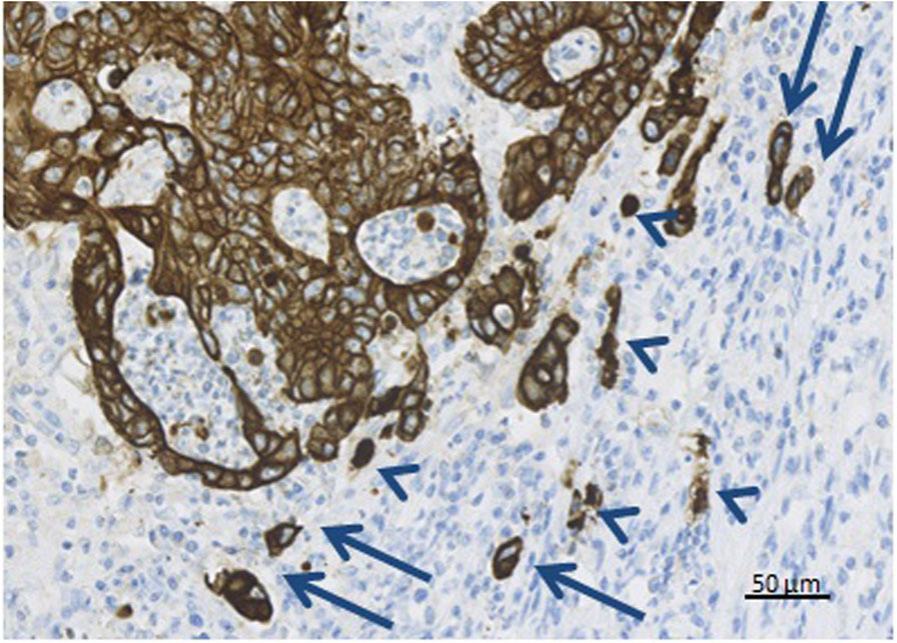
Counting tumour budding in a high power field (HPF). The number of tumour buds (TB) was counted along the invasive front on cytokeratin AE1/AE3 stained sections in 10 HPF. A tumour bud was defined as an isolated single tumour cell or a cluster of up to four tumour cells (arrows). Adenocarcinoma cells without a clear nucleus and cytoplasmatic fragments (arrowheads) were not counted. Magnification 40×

The number of tumour buds were counted along the invasive front using pan-cytokeratin (AE1/AE3) stained sections. First, the sections were examined at low magnification, and the area of the invasive margin representing the highest density of TB was subjectively selected (hot-spot sampling). The number of tumour buds were then counted in 10 HPFs using 40× objective with field size area 0.3 mm^2^ (Fig. 2). The first HPF was placed in the area with the highest budding density, and sampling of HPFs with the highest number of tumour buds were searched in both directions along the invasive tumour margin. A tumour bud was defined as an isolated, single adenocarcinoma cell or a small cluster of up to 4 tumour cells as defined by Ueno et al. [9]. Adenocarcinoma cells were excluded from the counts if they did not expose a clearly defined, blue stained nucleus to avoid counting immunohistochemically stained, brown cytoplasmic fragments and artefacts.

### Reproducibility of TSR and TB

Intra-observer analysis was performed on all sections for both TB and the semi-quantitative estimation of TSR with a four-week washout period as count A1 and A2, respectively. For inter-observer analysis a subset of 50 randomly selected sections was chosen, and TSR and TB were estimated by an independent observer as count B. The stereological, quantitative evaluation of TSR was not reproduced according to the high reliability of stereological methods.

### Statistical analysis

Data were summarized and inspected by standard statistical methods. Inter- and intra-tumoural variability was assessed by calculation of intra-class correlation coefficients (ICC) [21], using a mixed-effects model. In this setting ICC is a ratio of variances and considered as the percentage of the total variance accounted for by the differences among the tumours examined. The ICC will be high (ICC → 1) if the majority of the estimator variation is attributable to inter-tumoral variation, i.e. biological variation among the patients. In case the majority of variation is caused by intra-tumoral variation, i.e. heterogeneity, the ICC will be low (ICC → 0).

We used chi^2^-test to compare the estimates of TSR obtained by stereology and conventional microscopy. The correlations were evaluated by Spearman’s correlation coefficients, and the differences were tested using Wilcoxon signed rank test. The correlations of TB obtained in the deepest invasive section and the two randomly selected sections were evaluated by Pearson’s correlation coefficient, while Wilcoxon signed rank test was used to investigate differences in the mean number of buds between the three sections. Simple and weighted kappa (K) values were generated to compare intra- and inter-observer variability [22], and agreement was described according to Landis et al. [23] as moderate, substantial, and almost perfect for Κ values of 0.41–0.60, 0.61–0.80, and 0.81–1, respectively. All tests were two-sided and *P*-values less than 0.05 considered significant. The statistical analysis was performed using the software STATA version 14.0 (StataCorp, Texas, USA).

## Results

### Tumour stroma ratio

#### Comparison of stereological and semi-quantitative estimates of tumour stroma ratio

Using stereology the mean stromal fraction of all sections was 0.34 (range: 0.04–0.84) for the whole tumour area and 0.56 (range: 0.12–0.94) for the hot-spot sampling area. We found the semi-quantitative method to underestimate TSR compared to stereological estimates of the hot-spot sampled area (*p* < 0.001), and there was a trend towards overestimation of TSR compared to the stereological estimate within the whole tumour area (*p* = 0.291) (Additional file 1: Table S1).

The correlation between the semi-quantitative and stereological techniques was analyzed for all sections (*N* = 129) and for each of the three individual sections from each tumour, i.e. the section with the deepest tumour penetration and the two randomly selected sections A and B (Table 1). Except for random section B, the correlations were best for the hot-spot sampled areas, especially when based on the tissue section with the deepest tumour invasion.

**Table 1:** Correlation between estimates of tumuor stroma ratio obtained semi-quantitatively and by stereology

	Spearman’s correlation All sections (*n* = 129)	Spearman’s correlation Deepest section (*n* = 43)	Spearman’s correlation Random section A (*n* = 43)	Spearman’s correlation Random section B (*n* = 43)
Stereology (whole tumour area) and semi-quantitative method	0.424, *p* < 0.001	0.461, *p* < 0.002	0.230, *p* = 0.139	0.551, *p* < 0.001
Stereology (hot-spot sampled area) and semi-quantitative method	0.579, *p* < 0.001	0.621, *p* < 0.001	0.598, *p* < 0.001	0.501, *p* < 0.001

Semi-quantitative tumour stroma ratio was estimated by conventional microscopy. The stereological estimation was done either in the whole tumour area of the section or in the hot-spot sampled area. Correlation for all sections (*n* = 129) and for individual tumour sections (n = 43)

### Intra-tumoural heterogeneity of tumour stroma ratio

We found the ICCs obtained by the semi-quantitative method to be considerably higher than those obtained by stereology (Table 2). For the stereological estimates, we calculated ICCs, using both the continuous raw data and the ordinal data divided into the four groups and found similar results.

**Table 2:** Intra-class correlation coefficients (ICC) for estimates of tumour stroma ratio

	ICC	95% CI
Semi quantitative method A1	0.648	(0.533–0.762)
Semi quantitative method A2	0.611	(0.488–0.733)
Stereology (whole tumour area; continuous data)	0.592	(0.465–0.718)
Stereology (hot-spot sampled area; continuous data)	0.393	(0.236–0.550)
Stereology (whole tumour area; data categorized in 4 groups)	0.451	(0.301–0.601)
Stereology (hot-spot sampled area; data categorized in 4 groups)	0.322	(0.158–0.485)

Tumour stroma ratio (TSR) was estimated semi-quantitatively by conventional microscopy

Stereological TSR was estimated both in the whole available tumour area of the section and in the hot-spot sampled area

Abbreviations: *CI* confidence interval

Using the semi-quantitative method, we found a fairly good correlation between “the deepest invasive section” and the two randomly chosen sections A and B, respectively (Additional file 1: Table S2). Based on stereological estimates for the whole tumour area, the correlations were decreasing and they decreased further when using stereological estimates based on sampling in the hot-spot area.

### Intra- and inter-observer reproducibility of tumour stroma ratio

The intra-observer agreement for the semi-quantitative estimation of TSR was overall substantial (Kappa range 0.65–0.77) and improved to almost perfect (Kappa range 0.78–0.83), when categorizing the four tiered TSR-data into high (TSR > 50%) or low (TSR ≤ 50%). Also, the inter-observer agreement increased, when categorized into two groups (Kappa range 0.70–0.75) (Table 3).

**Table 3:** Intra-and inter-observer kappa values for semi-quantitative estimates of tumour stroma ratio

	N	Kappa values (TSR: 4-groups)	Kappa values (TSR: High/Low)	Weighted kappa (TSR: 4 groups)
TSR-A1 & TSR-A2	129	0.68, *p* < 0.001	0.83, *p* < 0.001	0.77, *p* < 0.001
TSR-A1 & TSR-A2	43	0.65, *p* < 0.001	0.78, *p* < 0.001	0.75, *p* < 0.001
TSR-A1 & TSR-B	50	0.45, *p* < 0.001	0.75, *p* < 0.001	0.64, *p* < 0.001
TSR-A2 & TSR-B	50	0.47, *p* < 0.001	0.70, *p* < 0.001	0.67, *p* < 0.001

Tumour stroma ratio was estimated semi-quantitatively by conventional microscopy. Intra-observer kappa values were calculated for all sections (*n* = 129) and the deepest invasive tumour section (*n* = 43), and inter-observer kappa values were calculated for 50 randomly selected sections. Kappa values were calculated for data expressed in four groups; TSR-4-groups (1: TSR > 75%, 2: 50% < TSR ≤ 75%, 3: 25% < TSR ≤ 50%, 4: TSR ≤ 25%) and data expressed in two groups; TSR high/low (high TSR = stroma ≤50% and low TSR: stroma > 50%)

Abbreviations: *TSR* tumour stroma ratio; *A1* First count by observer A; *A2* second count by observer A; *B* count by observer B

### Budding

#### Estimates of tumour budding

Including all sections we found a mean of 3.55 buds (range 0–32) *per* HPF, and the 50% percentile (median) was 2.7 buds *per* HPF. Using 10 buds *per* HPF as cut-off, we defined high-budding as an average of ≥10 buds across 10 HPFs, as proposed by Karamitopoulou et al. [18]. We found that 122 (94.6%) of the histological slides were classified as low-budding and seven (5.4%) as high-budding.

The mean number of buds *per* HPF was 3.5 for the sections representing the deepest invasive tumour margin and 3.8 and 3.3 for the randomly selected sections A and B, respectively. The Pearson correlation coefficients for the correlation between the deepest invasive tumour section and the random sections A or B ranged from 0.674 for the correlation between the former and random section A (count A1) to 0.812 for the correlation with random section B (count A2) (*p* < 0.001).

#### Heterogeneity of tumour budding

The magnitude of ICCs for TB was similar to that obtained for TSR (Table 4) and with similar results, when analyzed on the mean or the data converted into high- or low-budding. According to the calculated values of ICC, the majority of the variation is attributable to biological differences among the tumours. In count A1 the ICC of 0.646 means that 35.4% of the total variance is due to variation within the single tumour (heterogeneity and measurement noise). Thus, the intra-tumoural variation is considerably lower than the inter-tumoral variation.

**Table 4:** Intra-class correlation coefficient (ICC) for tumour budding

	ICC	95% CI
TB-A1	0.646	0.529–0.763
TB-A2	0.669	0.556–0.778
TB-A1 (cut-off 10)	0.650	0.536–0.764
TB-A2 (cut-off 10)	0.698	0.596–0.800

ICC is calculated for continuous data and for data divided into categories as low-budding (< 10 buds) and high-budding (≥ 10 buds) (*n* = 129)

Abbreviations: *CI* confidence interval; *TB* tumour budding; *A1* First count by observer A; *A2* second count by observer A

#### Intra- and inter-observer reproducibility of tumour budding

Overall, the intra-observer agreement was in a clinically useful range varying from moderate to substantial (Table 5). The kappa values increased when only considering the deepest invasive tumour section (Kappa = 0.79). The inter-observer agreement was moderate to substantial (Kappa range 0.46–0.73).

**Table 5:** Intra- and inter-observer agreement data for scoring of tumour budding

	N	Kappa values (Cut-off ≥10 buds)	Weighted kappa values (Continuous TB-scale)
TB-A1 & TB-A2	129	0.60, *p* < 0.001	0.70, *p* < 0.001
TB-A1 & TB-A2	43	0.79, *p* < 0.001	0.79, *p* < 0.001
TB-A1 & TB-B	50	0.46, *p* < 0.001	0.54, *p* < 0.001
TB-A2 & TB-B	50	0.73, *p* < 0.001	0.59, *p* < 0.001

Kappa was calculated for continuous data and for data divided into categories as low-budding and high-budding with a cut-off for high-budding of ≥10 buds. Intra-observer kappa was calculated for all sections (*n* = 129) and for the deepest invasive tumour section (*n* = 43). Inter-observer kappa was calculated for 50 randomly selected cases

Abbreviations: *TB* tumour budding; *A1* First count by observer A; *A2* second count by observer A; *B* count by observer B

## Discussion

In this methodological study we investigated the heterogeneity of TSR and TB in 43 consecutive stage II adenocarcinomas of the colon and found that the intra-tumoural variation of both TSR and TB was considerably lower than the biological variation. We also compared semi-quantitative estimates of TSR based on conventional microscopy with unbiased, stereological estimates and found a significant correlation between semi-quantitative estimates and stereological estimates in hot-spot sampled areas, especially when only considering the deepest invasive tumour section.

### Heterogeneity of tumour stroma ratio and tumour budding

We investigated the heterogeneity using three sections, well aware that this only represents a minor part of the whole tumour. Because of the retrospective design, it was impossible to overcome sampling bias, as the investigated tissue had already been sampled and prepared for diagnostic purposes. ICCs estimated by conventional microscopy for both TB and TSR are comparable. The similarity in intratumoral heterogeneity may reflect TB as the cell-biological correlate of the tumour stroma interphase.

We found ICCs for the stereological estimates of TSR to be considerably lower than the ICCs calculated for the semi-quantitative estimates. Moreover, the stereological estimates of TSR in the hot-spot sampled areas revealed a considerably lower ICC than those obtained from the whole tumour area. This reflects the differences in technical approach and may be explained by the higher degree of detail and precision afforded by stereology. The level of detail is highest in the stereological hot-spot sampled estimation of TSR, and as expected this results in the highest level of intratumoral heterogeneity.

### Comparison of stereological and semi-quantitative estimates of tumour stroma ratio

Stereology is considered the gold standard for obtaining quantitative, histopathological data, but only one other study has measured the relative proportion of tumour cells using virtual slides and point counting [6]. Comparison, however, was not made with any semi-quantitative method.

**Fig. 3:**
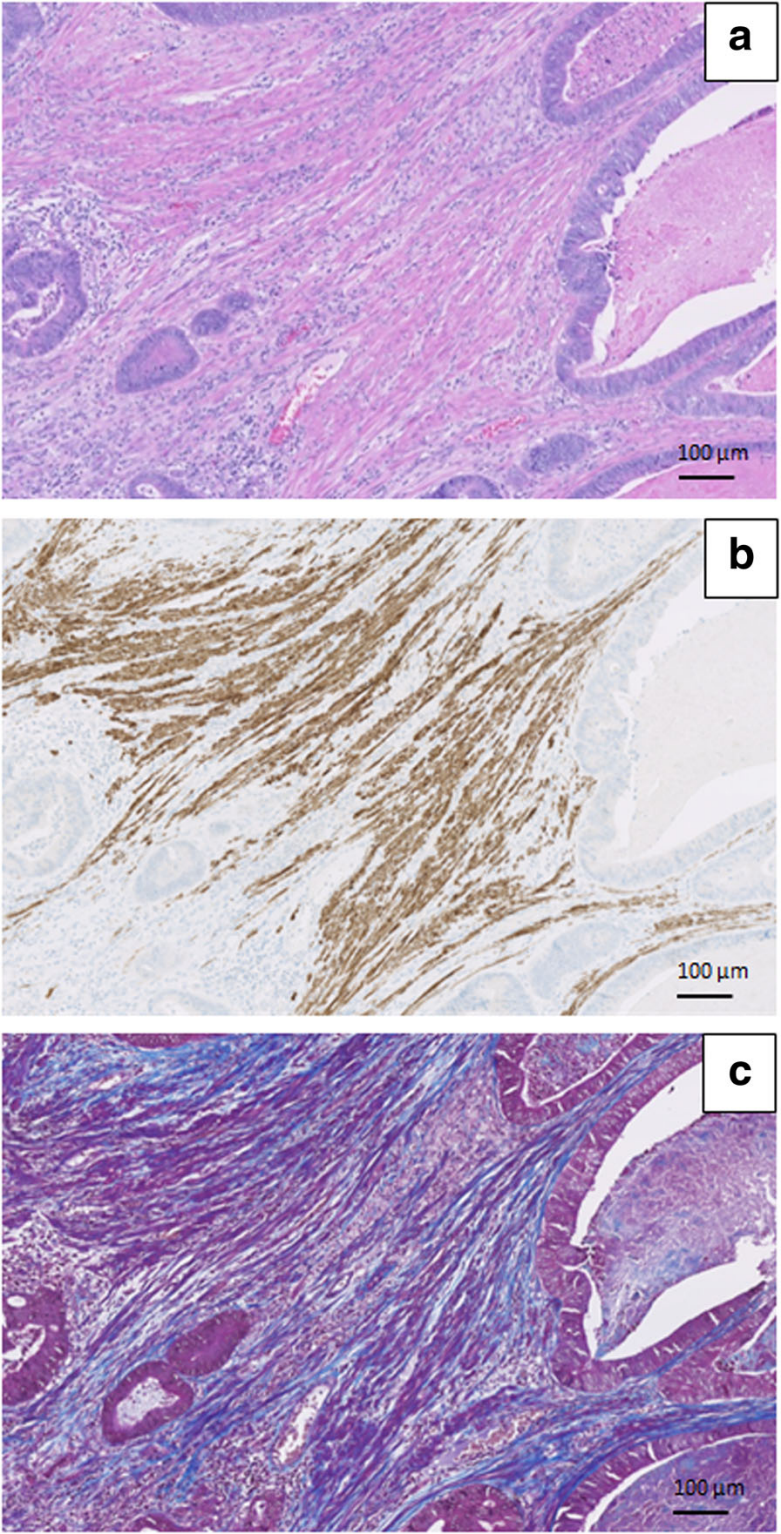
How to distinguish fibrous tumour stroma from smooth muscle tissue. **a** In the H&E stained section it is challenging to distinguish between fibrous stroma and smooth muscle tissue. **b** Immunohistochemical stain for desmin highlights the smooth muscle tissue. **c** Masson trichrome highlights the collagen fibres in the fibrous stroma (blue) and the smooth muscle tissue (purple-red). Magnification 10×

We found significant correlations for TSR obtained by stereology in the hot-spot sampled areas and by the semi-quantitative technique. Nevertheless, there were discrepancies. Part of the explanation might be the use of different study fields for the stereological hot-spot estimation and the semi-quantitative estimation, as these were not aligned but chosen subjectively for each method, guided by the highest stromal fraction. The ‘borderline’ cases were characterized by difficulty in distinguishing stroma and smooth muscle tissue. Morphologically, these two types of tissue can be distinguished by careful inspection of the cell nuclei. The smooth muscle cells have nuclei with rounded ends (cigar-shaped), while fibroblasts have more spindle shaped nuclei. Also, the smooth muscle fibres are often more eosinophilic in H&E stained sections than the collagenous, fibroblastic stroma. For some troublesome cases one may use Masson’s Trichrome, which stains smooth muscle fibres red and collagen fibres blue. Another option is immunohistochemical stain for desmin, which identifies smooth muscle fibres (Fig. 3). Both methods afford an easy distinguishing of fibrous stroma versus smooth muscle tissue.

Distinguishing fibrous stroma from smooth muscle tissue was problematic for both the stereological and the semi-quantitative approach. However, when in doubt using the semi-quantitative technique we had the possibility to find and compare the area of interest with the area of tunica muscularis, and the final decision was made “eye-balling” the overall representation of stroma in the whole tumour area. This was not an option for the stereological approach, and some variation was expected due to the subjectivity in deciding on fibrous stroma or smooth muscle. We have not investigated intra- and inter-observer variation for the stereological estimation, but studies using a similar approach report excellent inter-observer agreement with kappa values of 0.97 [6] and 0.986 [8]. Overall, stereological estimations have a high reproducibility due to the strict sampling and counting rules.

For the semi-quantitative method we found a moderate to substantial intra- and inter-observer agreement, which was optimized by using a dichotomized categorization. This is in accordance with previous studies using the same method, but results vary. In a recent study, TSR was evaluated using both a “global” method, estimating TSR in the whole available tumour area, and a “focal” method evaluating TSR in one single field (× 10 magnification) in the deepest invasive region of the tumour. The authors found method-dependent kappa values for intra-observer agreement of 0.45 (focal) and 0.84 (global), and the inter-observer kappa values were reported in the range 0.13–0.53 (focal) and 0.48–1.00 (global) [7]. The finding of the best TSR reproducibility being afforded by the “global” method contradicts earlier studies. A study using the “focal” method yielded an inter-observer kappa value of 0.89 [4], while studies using the “global” method reported kappa values between 0.60 and 0.70 [3, 24]. High inter-observer agreement on TSR has also been found in esophageal cancer based on the “focal” technical approach [25].

Estimation of TSR in mucinous tumours is debated. We investigated the impact of the mucinous component in the six mucinous adenocarcinomas included in our study by performing a sensitivity analysis excluding the sections of mucinous CC (*n* = 18). This resulted in almost unchanged correlation coefficients for correlations between the subjective, semi-quantitative estimates of TSR and the stereological estimates; 0.431 for whole section and 0.571 for hotspot area (*p* < 0.0001; data not shown).

Time consumption is a considerable disadvantage of stereological estimation of TSR. West et al [6] spent approximately 20 min *per* case and we spent on average 10 min *per* section. Thus, for routine use the stereological approach is considered too time consuming, whereas the semi-quantitative technique can be carried out in less than one minute. With optimal tissue stains and strict scoring criteria (Table 6), this method is reproducible and suited for the clinical setting.

**Table 6:** Scoring criteria for semi-quantitative estimation of tumour stroma ratio with a list of structures to be included or excluded in the estimation

Structure	Inclusion	Handling of problem
Necrosis	No	Select another area or another section if possible
Mucin	No	Select another area
Major vascular structures	No	Visual exclusion
Minor vascular structures	Yes	–
Smooth muscle tissue	No	Masson’s trichrome or desmin stains
Nerves	Yes	–
Lymphocytes	Yes	Dense infiltrates with neutrophil granulocytes are excluded as necrosis
Adenoma	No	Visual exclusion

### Estimates of tumour budding

Estimates of TB were obtained by the 10 HPFs method on pan-cytokeratin (AE1/AE3) stained sections as recommended in a recent review [26]. We found a considerable lower mean number of buds, and likewise lower proportion of high-budding tumours compared to other studies estimating TB on pan-cytokeratin stained sections [12, 27]; however the published studies are not comparable regarding both study population and techniques used. Horic M et al. [12] reported a higher median bud count of 8.05, but this study includes both colon and rectum cancer. Koelzer V H et al. [27] found a mean of 7.11 buds *per* HPF and 30.7% to be high-budding; however their cohorte also included upper rectal tumours. It is not described in the literature, whether the occurrence of TB in rectum cancers is comparable to TB in CC. Rectum tumours may represent a group with higher occurrence of TB than colon tumours, and in that case the two types of tumours must be considered separately according to TB.

Koelzer V H et al. [27] selected the section with the highest number of buds presented on H&E for IHC stain and evaluation of TB, while we used the deepest invasive section and further two randomly selected sections. In addition we only counted TB cells with a clearly identifiable nucleus to avoid cytoplasmic fragments etc., and our TB count might be lower due to this counting rule. Interestingly, we found inter-observer agreement similar to Koelzer V H et al., and thus the difference may exist in between centers. In regard to future studies using IHC, it is important to standardize this method of counting.

Overall we found acceptable intra- and inter-observer agreement, which is in accordance with earlier studies using the same method [17, 18]. However, a multicenter study found considerably lower reproducibility [28], but an explanation of this could be the selection of cases with only doubtful or controversial TB. We did not investigate TB using H&E, and thus we only discuss difficulties related to the use of IHC. We experienced differences in the definition of a clear nuclear stain and whether to count groups of tumour cells with TB appearance lying in between more solid areas, as these may represent cutting artefact of the cohesive 3D adenocarcinoma structure rather than real tumour buds. A disadvantage of the use of cytokeratin AE1/AE3 is the broad reactivity with staining of cell types other than the malignant adenocarcinoma cells. For instance, we experienced positive AE1/AE3 stain of mesothelial hyperplasia, which can be found in all serous membranes, and in some cases it was difficult to distinguish such cells from budding cells. A stain for cytokeratin 20 (CK 20) may distinguish budding cells from mesothelial hyperplasia, as the latter is negative for this particular cytokeratin. Unfortunately, we have experienced CK20 to be negative in approximately 10% of adenocarcinomas of the colon.

We also detected AE1/AE3 positive staining of endothelial cells of vascular neoangiogenesis, which could be difficult to distinguish from TB cells. However, strict criteria demanding a discernible nucleus in the tumour cell or a lumen in a vessel were helpful in most cases.

A number of other structures can have a budding-like appearance: fragmentation of tumour glands induced by abundant inflammatory infiltrate; retraction artifacts around fragmented tumour glands; fragments of tumour tissue surrounded by abundant mucinous extracellular matrix. Cytoplasmic fragments were also disturbing elements, but the strict rule of a visible tumour cell nucleus proved useful, as reported earlier in a multicenter study [17].

## Conclusion

This study of adenocarcinomas of the colon documents visual, subjective, semi-quantitative estimates of TSR to be correlated to stereological estimates of TSR with the best correlation afforded using the histological section with the deepest invasive tumour penetration. For both TSR and TB we found moderate to substantial inter-observer agreement, minor intra-tumoral heterogeneity, and high correlation among sections from the same tumour. For future studies of TSR we recommend the easy, cost efficient, and fast semi-quantitative technique and the use of one tissue section representing the deepest invasive tumour area of the adenocarcinoma. Regarding TB, we recommend using one tissue section. Considering the low proportion of high-budding tumours, further methodological studies are needed for evaluation of TB heterogeneity in rectal adenocarcinoma and CC in more advanced clinical stage of disease.

## Additional file

Additional file 1: **Table S1.** Tumour stroma ratio estimated semi-quantitatively by conventional microscopy and stereology. **Table S2.** Correlation coefficient for correlations between tumour stroma ratio in the deepest invasive tumour section and random sections A and B. (DOCX 40 kb)

## Abbreviations

AJCC: American Joint Committee
CC: Colon cancer
CK: Cytokeratin
CRC: Colorectal cancer
FFPE: Formalin fixed paraffin embedded
H&E: Hematoxylin and eosin
HPF: High power field
ICC: Intra-class correlation coefficient
NOS: Not otherwise specified
TB: Tumour budding
TSR: Tumour stroma ratio
