# Supplementary material for: Does heterogeneity matter in the estimation of tumour budding and tumour stroma ratio in colon cancer?
Source: Diagn Pathol. 2018 Mar 20;13:20. doi: 10.1186/s13000-018-0697-9 (PMC5859415; doi:10.1186/s13000-018-0697-9)
Supplement: Supplementary file 1 — Table S1. Tumour stroma ratio estimated semi-quantitatively by conventional microscopy and stereology. Table S2. Correlation coefficient for correlations between tumour stroma ratio in the deepest invasive tumour section and random sections A and B. (DOCX 40 kb) [file 13000_2018_697_MOESM1_ESM.docx]

**Additional file 1**

**Table S1** Tumour stroma ratio estimated semi-quantitatively by conventional microscopy and stereology

|  | **Stereology whole tumour area** | | | | **Stereology hotspot area** | | | | |
| --- | --- | --- | --- | --- | --- | --- | --- | --- | --- |
| **Semi quantitative**  **method** | **1** | **2** | **3** | **4** | **1** | **2** | **3** | **4** | **Total** |
| **1** | 27 | 17 | 4 | 0 | 6 | 23 | 19 | 0 | 48 |
| **2** | 20 | 36 | 3 | 0 | 3 | 14 | 35 | 7 | 59 |
| **3** | 5 | 10 | 5 | 1 | 0 | 2 | 9 | 10 | 21 |
| **4** | 0 | 0 | 0 | 1 | 0 | 0 | 1 | 0 | 1 |
| **Total** | 52 | 63 | 12 | 2 | 9 | 39 | 64 | 17 | 129 |

Tumour stroma ratio is divided in four groups as 1: TSR > 75%, 2: 50% < TSR ≤ 75%, 3: 25% < TSR ≤ 50%, 4: TSR ≤ 25% (n = 129).

**Table S2** Correlation coefficient for correlations between tumour stroma ratio in the deepest invasive tumour section and

random sections A and B

|  | **Deepest invasive tumour section** | | |
| --- | --- | --- | --- |
|  | **Semi quantitative**  **method** | **Stereology**  **(whole tumour area)** | **Stereology**  **(hot-spot sampled area)** |
| **Random section A** | 0.711, p<0.001 | 0.584, p<0.001 | 0.421, p= 0.005 |
| **Random section B** | 0.747, p<0.001 | 0.636, p<0.001 | 0.366, p=0.016 |

Spearman´s correlation is used for the semi-quantitative method and Pearson’s correlation for the

stereological estimates (n =43).
